# Updating the Genetic Landscape of Inherited Retinal Dystrophies

**DOI:** 10.3389/fcell.2021.645600

**Published:** 2021-07-13

**Authors:** Belén García Bohórquez, Elena Aller, Ana Rodríguez Muñoz, Teresa Jaijo, Gema García García, José M. Millán

**Affiliations:** ^1^Molecular, Cellular and Genomics Biomedicine, Health Research Institute La Fe, Valencia, Spain; ^2^CIBER of Rare Diseases, Madrid, Spain; ^3^Unit of Genetics, University Hospital La Fe, Valencia, Spain

**Keywords:** inherited retinal dystrophies, diagnosis, custom-panels, gene, pathogenic, deep-intronic

## Abstract

Inherited retinal dystrophies (IRD) are a group of diseases characterized by the loss or dysfunction of photoreceptors and a high genetic and clinical heterogeneity. Currently, over 270 genes have been associated with IRD which makes genetic diagnosis very difficult. The recent advent of next generation sequencing has greatly facilitated the diagnostic process, enabling to provide the patients with accurate genetic counseling in some cases. We studied 92 patients who were clinically diagnosed with IRD with two different custom panels. In total, we resolved 53 patients (57.6%); in 12 patients (13%), we found only one mutation in a gene with a known autosomal recessive pattern of inheritance; and 27 patients (29.3%) remained unsolved. We identified 120 pathogenic or likely pathogenic variants; 30 of them were novel. Among the cone-rod dystrophy patients, *ABCA4* was the most common mutated gene, meanwhile, *USH2A* was the most prevalent among the retinitis pigmentosa patients. Interestingly, 10 families carried pathogenic variants in more than one IRD gene, and we identified two deep-intronic variants previously described as pathogenic in *ABCA4* and *CEP290*. In conclusion, the IRD study through custom panel sequencing demonstrates its efficacy for genetic diagnosis, as well as the importance of including deep-intronic regions in their design. This genetic diagnosis will allow patients to make accurate reproductive decisions, enroll in gene-based clinical trials, and benefit from future gene-based treatments.

## Introduction

Inherited retinal dystrophies (IRD) are a group of diseases characterized by the progressive death or dysfunction of photoreceptors that leads to vision loss and, in some cases, legal blindness. IRDs have a prevalence of one case in 3,000 individuals (Sahel et al., 2015). Depending on the photoreceptor initially affected, IRD can be classified into cone, rod-cone, or cone-rod dystrophies in those cases in which both are affected at one time. Moreover, they can manifest as either isolated (70–80% of the total) or part of one of the 80 syndromes that have been estimated to be associated with IRD (Ayuso and Millan, 2010; Tatour and Ben-Yosef, 2020).

This group of diseases has a wide clinical spectrum and number of involved genes, currently reaching 271 for syndromic and non-syndromic forms (Retnet^1^, December 2020); explaining why IRDs have such a high clinical and genetic heterogeneity. Furthermore, there is a high inter and intrafamily variability, variable expression, and incomplete penetrance (Farrar et al., 2017). IRDs can follow different patterns of inheritance, including autosomal recessive, autosomal dominant, X-linked, mitochondrial mode, and some other less common, such as uniparental isodisomy or digenic inheritance (Kajiwara et al., 1994; Dryja et al., 1997; Rivolta et al., 2002; Ayuso and Millan, 2010; Parmeggiani et al., 2011; Neveling et al., 2012). These issues, together with the fact that 50% are sporadic cases, make it even more complicated to determine the mode of inheritance, genetic diagnosis, and genetic counseling (Perea-Romero et al., 2021).

With the advent of next generation sequencing (NGS), the ratio of diagnosis has risen to 50–70%, which was difficult to achieve a few years ago (Carss et al., 2017; Sanchis-Juan et al., 2018; Rodríguez-Muñoz et al., 2020). Some different approaches, such as custom panel designs, whole exome sequencing (WES), and whole genome sequencing (WGS), have been implemented to study the molecular mechanisms of IRD. Currently, these new sequencing techniques are essential to obtain an early and accurate genetic diagnosis, which is necessary to offer a correct genetic counseling to patients and their families (Salmaninejad et al., 2019).

In this study, we analyzed 92 patients, who were previously clinically diagnosed with IRD, with two custom panels for the main aim of achieving genetic diagnoses.

## Materials and Methods

### Cohort Selection

We selected 92 patients, belonging to 90 different Spanish families, with a clinical diagnosis of non-syndromic IRD, except for one who had a clinical suspicion of a syndromic IRD. Patient DNA was isolated from peripheral blood with the automatic extractor QIAsymphony (QIAGEN).

Patients underwent complete ophthalmologic examinations, including OCT (Heidelberg Spectralis OCT Bluepeak, Heidelberg, Germany; Topcon 3D OCT 2000, Tokyo, Japan; CIRRUS OCT Zeiss, Oberkochen, Germany), ERG (Roland RETI-port/scan21, Brandenburg, Germany), eye fundus (Visucam NM/FA Zeiss, Oberkochen, Germany), visual acuity measure (BCVA), and evoked potentials and visual fields (Carl Zeiss Humphrey Field Analyzer, Oberkochen, Germany). A clinical questionnaire, which collected the main IRD characteristics, and an informed consent were completed by each patient. This study was approved by the Hospital La Fe Ethics Committee, in agreement with the Declaration of Helsinki.

### Panel Design and Sequencing

Sixty-three patients were sequenced with a gene panel version (PV1) that included 117 genes involved in a non-syndromic IRD, and their flanking intronic regions (±25 base pairs) (Rodríguez-Muñoz et al., 2020). Moreover, the panel of genes contained five intronic regions of *ABCA4*, *OFD1*, *USH2A*, *CEP290*, and *PRPF31*, in which pathogenic variants had been previously identified (Den Hollander et al., 2006; Littink et al., 2010; Vaché et al., 2012; Webb et al., 2012; Braun et al., 2013; Supplementary Table 1).

The remaining 29 cases were analyzed with an updated version of the custom panel (PV2) that had 114 genes and all the deep-intronic variants described in the last few years in *ABCA4* and *USH2A* (Vaché et al., 2012; Braun et al., 2013; Zernant et al., 2014; Bauwens et al., 2015, 2019; Liquori et al., 2016; Baux et al., 2017; Fadaie et al., 2019; Khan et al., 2019; Sangermano et al., 2019; Supplementary Table 2).

The patients’ libraries were prepared in accordance with the SureSelect QXT protocol (Agilent Technologies) and sequenced on a MiSeq platform (Illumina, San Diego, CA) in 300 cycles with 2 × 150 base pairs reads.

### Data Analysis

The reads alignment against the reference hg19 genome, variant calling, and annotation of all the identified variants were carried out with the *Alissa* resource (Agilent Technologies). The obtained variants were filtered based on a MAF (minor allele frequency) ≤ 0.01 according to the ExAC^2^ and gnomAD^3^ databases. In order to evaluate the pathogenicity of the detected variants, we also evaluated specific databases such as ClinVar^4^, Locus Specific Data Base^5^, and HGMD professional^6^. To evaluate the potential effect of novel missense variants, we used the *in silico* predictors included in Varsome^7^. The putative effect on the splicing process was performed with HSF (Human SplicingFinder^8^), NNSplice^9^, and SpliceAI^10^. Finally, the IGV view finder^11^ allowed the examination of all detected variants in every read.

The novel variants identified in this study were classified according to the standards of the American College of Medical Genetics and Genomics (ACMG) (Richards et al., 2015).

### Copy Number Variation Analysis

A copy number variations (CNV) analysis was performed using the DECoN bioinformatic tool version 1.0.2 (Fowler et al., 2016).

We also studied large rearrangements by Multiplex Ligation-dependent Probe Amplification (MLPA; MRC Holland) in patients harboring one pathogenic variant in *USH2A* (probemixes P361 and P362), *EYS* (probemix P328-A3), and*ABCA4* (probemixes P151 and P152). The multiplex ligation-dependent probe amplification results were analyzed by the Coffalyser. Net software version 140721.1958 (MRC-Holland).

### Sanger Sequencing and Segregation Analysis

The candidate variants identified in each patient were validated by Sanger sequencing (Big Dye Terminator v1.1 or v3.1, Applied Biosystems by Life Technologies). Moreover, the deep-intronic variants, already described as pathogenic in *USH2A* and *ABCA4* (Supplementary Table 3; Vaché et al., 2012; Braun et al., 2013; Zernant et al., 2014; Bauwens et al., 2015, 2019; Liquori et al., 2016; Baux et al., 2017; Fadaie et al., 2019; Khan et al., 2019; Sangermano et al., 2019), were studied by Sanger sequencing in patients analyzed with PV1 who carried only one pathogenic variant in either or both genes.

Segregation analysis was performed when relatives’ DNA was available.

## Results

We obtained a mean depth of 190× per patient. In 98.6% of the patients, at least 88% of the bases were covered with a sequencing depth of coverage ≥50× and 95% of the bases were covered with at least 20×. Moreover, we obtained a 70% of on-target reads.

We solved 53 patients belonging to 52 families (53/92, ratio of 57.6%): 17 (of 17 families) which had disease-causing variants in autosomal dominant genes, 33 (of 32 families) with autosomal recessive genes, two patients (of two families) who carried a pathogenic variant in an X-linked gene and one patient who carried two different pathogenic variants in two different autosomal dominant genes and two additional variants in an autosomal recessive gene (RPN-670). We reported five cases with more than two pathogenic variants in the same autosomal recessive gene, and we also described six patients who carry pathogenic variants in more than one IRD gene (Table 1). Among the unresolved patients, 13% carried one pathogenic variant in an autosomal recessive IRD gene, with a higher prevalence of cases with a heterozygous variant in *ABCA4*, followed by *USH2A* and *RPGRIP1* (Table 2). In the remaining 29.3% cases, no pathogenic variant was identified.

**TABLE 1:** Pathogenic variants identified in solved patients.

Family	Patient	Clinical diagnosis	Gene	Nucleotide change	Protein change	Zygosity	References
$FRPN-51^1^	RPN-129	STG	*ABCA4* (NM_000350.3)	c.1804C > T	p.(Arg602Trp)	Heterozygous	Lewis et al., 1999
				c.982G > T	p.(Glu328*)	Heterozygous	Fishman, 2003
	RPN-544	STG	*ABCA4* (NM_000350.3)	c.5882G > A	p.(Gly1961Glu)	Heterozygous	Allikmets et al., 1997
				c.982G > T	p.(Glu328*)	Heterozygous	Fishman, 2003
$FRPN-244^1^	RPN-646	RP	*RHO* (NM_000539.3)	c.328T > C	p.(Cys110Arg)	Heterozygous	To et al., 2004
FRPN-246^1^	RPN-649	MD/STG	*ABCA4* (NM_000350.3)	c.5917del	p.(Val1973*)	Homozygous	Rivolta et al., 2000
$FRPN-252^1^	RPN-657	STG	*ABCA4* (NM_000350.3)	**c.5714 + 1G > A**	**p.?**	**Heterozygous**	**This study**
				c.3386G > T	p.(Arg1129Leu)	Heterozygous	Allikmets et al., 1997
FRPN-254^1^	RPN-659	RP	*EYS* (NM_001142800.2)	**c.7736_7742del**	p.(Thr2579Lysfs*36)	**Homozygous**	**This study**
FRPN-255^1^	RPN-660	RP	*USH2A* (NM_206933.4)	c.4732C > T	p.(Arg1578Cys)	Heterozygous	Le Quesne Stabej et al., 2012
				c.1214del	p.(Asn405Ilefs*3)	Heterozygous	Schwartz et al., 2005
FRPN-256^1^	RPN-661	RP/LCA	*CRB1* (NM_201253.3)	c.2416G > T	p.(Glu806*)	Homozygous	Corton et al., 2013
FRPN-258^1^	RPN-663	STG	*ABCA4* (NM_000350.3)	c.3386G > T	p.(Arg1129Leu)	Homozygous	Allikmets et al., 1997
				c.6718A > G	p.(Thr2240Ala)	Heterozygous	Zernant et al., 2011
FRPN-261^1^	RPN-666	Reverse BCAMD/STG/RP	*ABCA4* (NM_000350.3)	c.5929G > A	p.(Gly1977Ser)	Heterozygous	Rozet et al., 1998
				c.5882G > A	p.(Gly1961Glu)	Heterozygous	Allikmets et al., 1997
FRPN-263^1^	RPN-668	CD	*CRB1* (NM_201253.3)	c.1604T > C	p.(Leu535Pro)	Heterozygous	Corton et al., 2013
				c.2843G > A	p.(Cys948Tyr)	Heterozygous	den Hollander et al., 1999
FRPN-265^1^	RPN-670	NA	*RP1* (NM_006269.2)	c.3157del	p.(Tyr1053Thrfs*4)	Heterozygous	Jacobson et al., 2000
			*PRPH2* (NM_000322.5)	c.623G > A	p.(Gly208Asp)	Heterozygous	Kohl et al., 1997
			*USH2A* (NM_206933.4)	**c.6957 + 1G > C**	**p.?**	**Heterozygous**	**This study**
				**c.4955C > T**	**p.(Pro1652Leu)**	**Heterozygous**	**This study**
FRPN-266^1^	RPN-671	RP	*EYS* (NM_001142800.2)	c.5928-2A > G	p.?	Homozygous	González-del Pozo et al., 2011
FRPN-267^1^	RPN-672	RP	*CNGB1* (NM_001297.5)	**c.2492 + 2T > G**	**p.?**	**Homozygous**	**This study**
FRPN-268^1^	RPN-673	RP	*USH2A* (NM_206933.4)	c.2276G > T	p.(Cys759Phe)	Heterozygous	Rivolta et al., 2000
				**c.13894C > T**	**p.(Pro4632Ser)**	**Heterozygous**	**This study**
FRPN-269^1^	RPN-675	RP	*USH2A* (NM_206933.4)	c.4732C > T	p.(Arg1578Cys)	Heterozygous	Le Quesne Stabej et al., 2012
				c.12575G > A	p.(Arg4192His)	Heterozygous	Ávila-Fernández et al., 2010
FRPN-273^1^	RPN-679	STG	*ABCA4* (NM_000350.3)	**c.4880del**	p.(Leu1627Argfs*35)	**Heterozygous**	**This study**
				c.5714 + 5G > A	p.?	Heterozygous	Cremers, 1998
				**c.2953G > A**	**p.(Gly985Arg)**	**Heterozygous**	**This study**
FRPN-276^1^	RPN-682	MD	*BEST1* (NM_004183.4)	c.247G > T	p.(Val83Phe)	Heterozygous	Kinnick et al., 2011
FRPN-277^1^	RPN-683	RP	*RPGR* (NM_001034853.2)	**c.1366del**	p.(Gln456Lysfs*20)	**Hemizygous**	**This study**
FRPN-278^1^	RPN-684	RP	*EYS* (NM_001142800.2)	c.9468T > A	p.(Tyr3156*)	Homozygous	Collin et al., 2008
FRPN-279^1^	RPN-685	NA	*PRPH2* (NM_000322.5)	c.658C > T	p.(Arg220Trp)	Heterozygous	Payne et al., 1998
FRPN-280^1^	RPN-686	RP	*USH2A* (NM_206933.4)	c.12575G > A	p.(Arg4192His)	Homozygous	Ávila-Fernández et al., 2010
FRPN-281^1^	RPN-687	CRD/STG	*CRB1* (NM_201253.3)	**c.481G > A**	**p.(Ala161Thr)**	**Homozygous**	**This study**
FRPN-284^1^	RPN-692	RP Punctata albensces	*ABCA4* (NM_000350.3)	c.3386G > T	p.(Arg1129Leu)	Heterozygous	Allikmets et al., 1997
				c.6148G > C	p.(Val2050Leu)	Heterozygous	Lewis et al., 1999
$FRPN-286^1^	RPN-694	NA	*PRPH2* (NM_000322.5)	**c.440dup**	p.(Gly148Trpfs*29)	**Heterozygous**	**This study**
$FRPN-289^1^	RPN-697	STG	*ABCA4* (NM_000350.3)	c.3386G > T	p.(Arg1129Leu)	Heterozygous	Allikmets et al., 1997
				c.634C > T	p.(Arg212Cys)	Heterozygous	Gerber et al., 1998
FRPN-293^1^	RPN-701	RP	*PRPH2* (NM_000322.5)	**c.647C > A**	**p.(Pro216His)**	**Heterozygous**	**This study**
FRPN-294^1^	RPN-702	MD	*ELOVL4* (NM_022726.4)	c.59A > G	p.(Asn20Ser)	Heterozygous	Hu et al., 2020
FRPN-296^1^	RPN-704	CD	*GUCA1A* (NM_000409.5)	**c.66C > A**	p.(Tyr22*)	**Heterozygous**	**This study**
FRPN-298^1^	RPN-706	CRD	*CRB1* (NM_201253.3)	c.613_619del	p.(Ile205Aspfs*13)	Homozygous	Lotery, 2001
				c.2291G > A	p.(Arg764His)	Heterozygous	Corton et al., 2013
FRPN-299^1^	RPN-707	RP	*USH2A* (NM_206933.4)	c.13811 + 2T > G	p.?	Heterozygous	Besnard et al., 2014
				c.2276G > T	p.(Cys759Phe)	Heterozygous	Rivolta et al., 2000
FRPN-300^1^	RPN-708	NA	*OTX2* (NM_001270525.2)	**c.638T > A**	p.(Leu213*)	**Heterozygous**	**This study**
FRPN-301^1^	RPN-709	RP	*PROM1* (NM_006017.2)	**deletion exon 11**	**p.?**	**Homozygous**	**This study**
FRPN-302^1^	RPN-710	MD/BVMD	*PRPH2* (NM_000322.5)	c.641G > A	p.(Cys214Tyr)	Heterozygous	Trujillo et al., 2001
FRPN-303^1^	RPN-711	RP	*USH2A* (NM_206933.4)	c.14803C > T	p.(Arg4935*)	Heterozygous	Baux et al., 2007
				c.2332G > T	p.(Asp778Tyr)	Heterozygous	Lenassi et al., 2015
FRPN-307^1^	RPN-715	RP	*RHO* (NM_000539.3)	c.512C > A	p.(Pro171Gln)	Heterozygous	Antiñolo et al., 1994
$FRPN-308^2^	RPN-717	RP	*ABCA4* (NM_000350.3)	c.6148G > C	p.(Val2050Leu)	Heterozygous	Allikmets et al., 1997
			*PRPF31* (NM_015629.3)	**Gene deletion**	**p.?**	**Heterozygous**	**This study**
FRPN-309^2^	RPN-718	RP	*USH2A* (NM_206933.4)	c.2276G > T	p.(Cys759Phe)	Homozygous	Rivolta et al., 2000
FRPN-312^2^	RPN-721	RP	*RHO* (NM_000539.3)	c.512C > A	p.(Pro171Leu)	Heterozygous	Stone et al., 2017
FRPN-315^2^	RPN-725	MD/STG	*ABCA4* (NM_000350.3)	**c.6310C > T**	p.(Gln2104*)	**Heterozygous**	**This study**
				c.3386G > T	p.(Arg1129Leu)	Heterozygous	Allikmets et al., 1997
FRPN-316^2^	RPN-726	RP	*NRL* (NM_001354768.3)	c.149C > T	p.(Ser50Leu)	Heterozygous	Koyanagi et al., 2019
			*ABCA4* (NM_000350.3)	c.5908C > T	p.(Leu1970Phe)	Heterozygous	Rozet et al., 1998
FRPN-318^2^	RPN-728	Joubert syndrome	*CEP290* (NM_025114.4)	c.4966_4967del	p.(Glu1656Asnfs*3)	Heterozygous	Sheck et al., 2018
				c.2817G > T	p.(Lys939Asn)	Heterozygous	Srivastava et al., 2017
FRPN-320^2^	RPN-730	MD/STG	*PRPH2* (NM_000322.5)	c.537G > A	p.(Trp179*)	Heterozygous	Diñeiro et al., 2020
FRPN-322^2^	RPN-732	RP	*PDE6B* (NM_000283.4)	**c.1920 + 1G > A**	**p.?**	**Heterozygous**	**This study**
				**c.2470_2478del**	**p.(Lys824_Glu826del)**	**Heterozygous**	**This study**
FRPN-323^2^	RPN-733	MD	*PRPH2* (NM_000322.5)	c.421T > C	p.(Tyr141His)	Heterozygous	Diñeiro et al., 2020
			*ABCA4* (NM_000350.3)	c.5908C > T	p.(Leu1970Phe)	Heterozygous	Rozet et al., 1998
FRPN-324^2^	RPN-734	MD/CD	*ABCA4* (NM_000350.3)	c.3113C > T	p.(Ala1038Val)	Heterozygous	Rozet et al., 1998
				c.4539 + 2064C > T	[p.?; p.(=, Arg1514Leufs*36)]	Heterozygous	Zernant et al., 2014; Bauwens et al., 2019
				c.1364T > A	p.(Leu455Gln)	Heterozygous	Salles et al., 2018
FRPN-325^2^	RPN-735	NA	*EYS* (NM_001142800.2)	**c.8854del**	p.(Thr2973Leufs*23)	**Heterozygous**	**This study**
				**c.1194del**	p.(Gly399Aspfs*22)	**Heterozygous**	**This study**
			*RDH5* (NM_002905.3)	c.712G > T	p.(Gly238Trp)	Heterozygous	Gonzalez-Fernandez et al., 1999
FRPN-328^2^	RPN-737	MD/STG	*PRPH2* (NM_000322.5)	c.641G > A	p.(Cys214Tyr)	Heterozygous	Trujillo et al., 2001
FPRN-327^2^	RPN-738	MD/STG	*BBS1* (NM_024649.4)	c.1169T > G	p.(Met390Arg)	Homozygous	Nishimura et al., 2010
FRPN-335^2^	RPN-745	RP	*RPGR* (NM_001034853.2)	c.935-2A > G	p.?	Hemizygous	Koyanagi et al., 2019
			*ABCA4* (NM_000350.3)	c.5908C > T	p.(Leu1970Phe)	Heterozygous	Rozet et al., 1998
			*PDE6A* (NM_000440.3)	**c.2144T > C**	**p.(Met715Thr)**	**Heterozygous**	**This study**
FRPN-337^2^	RPN-747	RP	*RHO* (NM_000539.3)	**c.670G > A**	**p.(Gly224Arg)**	**Heterozygous**	**This study**
FRPN-339^2^	RPN-749	STG	*ABCA4* (NM_000350.3)	c.3386G > T	p.(Arg1129Leu)	Heterozygous	Allikmets et al., 1997
				c.3210_3211dup	p.(Ser1071Cysfs*14)	Heterozygous	Nasonkin et al., 1998
				c.560G > A	p.(Arg187His)	Heterozygous	Cornelis et al., 2017
$FRPN-340^2^	RPN-750	LCA	*CEP290* (NM_025114.4)	c.2991 + 1655A > G	p.?	Homozygous	Den Hollander et al., 2006

*Novel pathogenic variants are highlighted in bold font.*

FRPN, family number; FRPN-“^1,^” families studied with PV1; FRPN-“^2,^” families studied with PV2; $FRPN, families in which segregation analysis was performed; RPN, patient number; RP, retinitis pigmentosa; MD, macular dystrophy; p.?, unknown protein effect; STG, stargardt; LCA, leber congenital amaurosis; BCAMD, benign concentric annular macular dystrophy; CD, cone dystrophy; CRD, cone-rod dystrophy; BVMD, best vitelliform macular dystrophy; NA, not available. The “*” symbol corresponds to: stop-codon according to the format of mutations nomenclature from the Human Genome Variation Society (HGVS).

**TABLE 2:** Patients in which only one pathogenic variant in a recessive gene has been identified.

Family	Patient	Clinic diagnosis	Gene	Nucleotide change	Protein change	Zygosity	References
FRPN-243^1^	RPN-645	RP	*USH2A* (NM_206933.4)	c.2276G > T	p.(Cys759Phe)	heterozygous	Rivolta et al., 2000
			*CEP290* (NM_025114.4)	**c.7394_7395del**	p.(Glu2465Valfs*2)	**heterozygous**	**This study**
FRPN-272^1^	RPN-678	STG	*ABCA4* (NM_000350.3)	c.288C > A	p.(Asn96Lys)	heterozygous	Stenirri et al., 2004
			*USH2A* (NM_206933.4)	c.754G > T	p.(Gly252Cys)	heterozygous	Bravo-Gil et al., 2016
FRPN-283^1^	RPN-691	RP Punctata albensces	*CDHR1* (NM_033100.3)	c.783G > A	(p.Pro261 =)	heterozygous	Glockle et al., 2014
			*POC1B* (NM_172240.3)	**c.1079_1080del**	p.(Pro360Argfs*8)	**heterozygous**	**This study**
FRPN-287^1^	RPN-695	NA	*ABCA4* (NM_000350.3)	c.6089G > A	p.(Arg2030Gln)	heterozygous	Lewis et al., 1999
FRPN-288^1^	RPN-696	NA	*ABCA4* (NM_000350.3)	c.6148G > C	p.(Val2050Leu)	heterozygous	Allikmets et al., 1997
FRPN-304^1^	RPN-712	RP	*RPGRIP1* (NM_020366.3)	**c.3339 + 5G > C**	**p.?**	**heterozygous**	**This study**
FRPN-305^1^	RPN-713	RP	*SAG* (NM_000541.5)	c.577C > T	p.(Arg193*)	heterozygous	Maw et al., 1998
			*GUCY2D* (NM_000180.4)	**c.1991A > G**	**p.(His664Arg)**	**heterozygous**	**This study**
FRPN-308^2^	RPN-717	RP	*ABCA4* (NM_000350.3)	c.6148G > C	p.(Val2050Leu)	heterozygous	Allikmets et al., 1997
FRPN-311^2^	RPN-720	RP (early onset)	*RPGRIP1* (NM_020366.3)	c.767C > G	p.(Ser256*)	heterozygous	Jamshidi et al., 2019
FRPN-313^2^	RPN-722	RP	*RBP3* (NM_002900.3)	c.3238G > A	p.(Asp1080Asn)	heterozygous	den Hollander et al., 2009
FRPN-314^2^	RPN-724	RP	*EYS* (NM_001142800.2)	**c.6882_6883del**	p.(Gln2294Hisfs*3)	**heterozygous**	**This study**
FRPN-319^2^	RPN-729	RP	*CNGA3* (NM_001298.3)	**c.673 + 5G > T**	**p.?**	**heterozygous**	**This study**
FRPN-336^2^	RPN-746	MD	*PCARE* (NM_001271441.2)	**c.656_665dup**	p.(Ala223Argfs*38)	**heterozygous**	**This study**

*Novel pathogenic variants identified are highlighted in bold font.*

FRPN, family number; FRPN-“^1,^” families studied with PV1; FRPN-“^2,^” families studied with PV2; RPN, patient number; RP, retinitis pigmentosa; STG, stargardt; NA, not available; p.?, unknown protein effect; SNHL, sensorineural hearing loss; XL, X-linked. The “*” symbol corresponds to: stop-codon according to the format of mutations nomenclature from the Human Genome Variation Society (HGVS).

**FIGURE 1:**
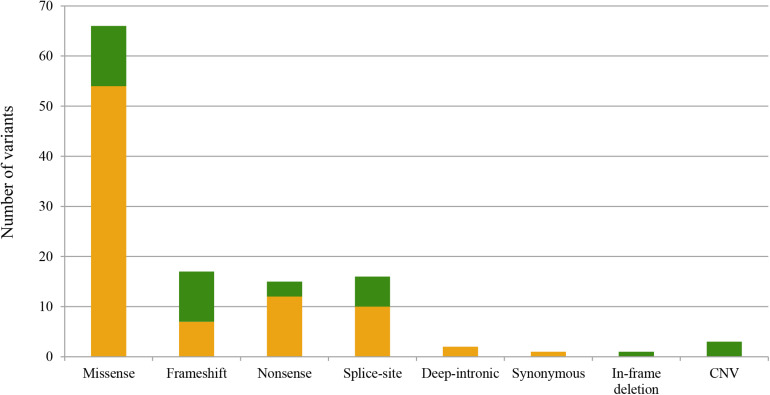
Representation of the total alleles identified in this study classified according to the alteration type. The X-axis refers to the different types of variants identified in this study, and the Y-axis concerns the total allele count for each type of variant. Pathogenic variants identified in this study are represented in green, while those previously described are in orange.

**FIGURE 2:**
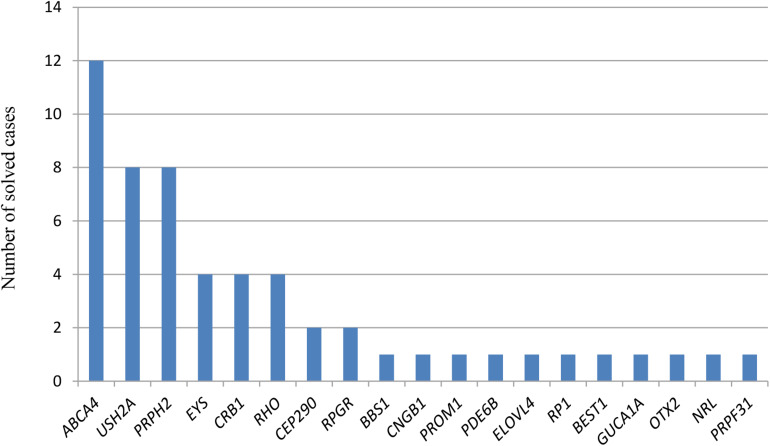
Number of solved cases with mutations in the different disease-causing genes. The X-axis refers to the responsible genes for each solved case, and the Y-axis to the number of solved cases for each disease-causing gene. Only genes responsible for the disease are represented. For patient RPN-670 (FRPN-265), the three different possible responsible genes for the IRD are represented.

We identified a total of 120 pathogenic or likely pathogenic variants (of which, 85 were unique), including: 65 missense, 15 nonsense, 17 frameshift, 16 splice-site variants, two pathogenic deep-intronic variants, one in-frame deletion, one synonymous, and three CNV (Figure 1). Twenty-nine variants were first described in this study (Tables 1–3). In line with this, we identified a high number of mutated-genes, nine autosomal dominant genes (highest prevalence of *PRPH2*); 19 autosomal recessive genes, among which, *ABCA4* and *USH2A* stand out; and finally, one X-linked gene, *RPGR* (Figure 2). All identified novel variants were classified as pathogenic or likely pathogenic according to the ACMG criteria except for variant c.2470_2478del (p.Lys824_Glu826del) in *PDE6B*, which remained as a variant of uncertain significance. This patient harbored another heterozygous pathogenic variant in the same gene; however, we could not carry out the segregation analysis to confirm it as likely pathogenic.

**TABLE 3:** Criteria considered for the pathogenicity classification of the novel identified variants.

Gene	Mutation		Classification	Frequency (gnomAD Ex)	Pathogenicity scores^a^	Conservation score (GERP)^b^	Reputable source	
	**Nucleotide change**	**Protein change**					**ClinVar**	**HGMD^c^**
*ABCA4* (NM_000350.3)	c.2953G > A	p.(Gly985Arg)	Likely pathogenic	NF	12 of 13	5.7199	NA	NA
	c.4880del	p.(Leu1627Argfs*35)	Pathogenic	0.0000159	NA	5.6399	Pathogenic	NA
	c.5714 + 1G > A	p.?	Pathogenic	0.00000398	NA	4.7399	Likely pathogenic	NA
	c.6310C > T	p.(Gln2104*)	Pathogenic	NF	NA	6.0799	NA	NA
*CEP290* (NM_025114.4)	c.7394_7395del	p.(Glu2465Valfs*2)	Pathogenic	0.000598	NA	5.42	NA	NA
*CNGA3* (NM_001298.3)	c.673 + 5G > T	p.?	Likely pathogenic	0.0000199	NA	5.09	NA	NA
*RPGRIP1* (NM_020366.3)	c.3339 + 5G > C	p.?	Likely pathogenic	NF	2 of 2	NA	Likely pathogenic	NA
*CNGB1* (NM_001297.5)	c.2492 + 2T > G	p.?	Pathogenic	NF	NA	5.4499	NA	NA
*CRB1* (NM_201253.3)	c.481G > A	p.(Ala161Thr)	Likely pathogenic	NF	12 of 13	5.5199	Uncertain significance	NA
*PCARE* (NM_001271 441.2)	c.656_665dup	p.(Ala223Argfs*38)	Likely pathogenic	0.0000579	NA	1.8514	NA	NA
*EYS* (NM_0011 42800.2)	c.1194del	p.(Gly399Aspfs*22)	Pathogenic	NF	NA	6.07	NA	NA
	c.6882_6883del	p.(Gln2294Hisfs*3)	Pathogenic	NF	NA	5.38	NA	NA
	c.7736_7742del	p.(Thr2579Lysfs*36)	Pathogenic	0.0000127	NA	4.01	Likely pathogenic	NA
	c.8854del	p.(Thr2952Leufs*23)	Pathogenic	NF	NA	4.57	NA	NA
*GUCY2D* (NM_000180.4)	c.1991A > G	p.(His664Arg)	Likely pathogenic	NF	11 of 12	5.35	NA	NA
*GUCA1A* (NM_000409.5)	c.66C > A	p.(Tyr22*)	Likely pathogenic	0.00000795	NA	5.75	Uncertain significance	NA
*OTX2* (NM_001 270525.2)	c.638T > A	p.(Leu213*)	Pathogenic	NF	NA	5.53	NA	NA
*PDE6A* (NM_000440.3)	c.2144T > C	p.(Met715Thr)	Likely pathogenic	0.000231	12 of 13	5.32	Uncertain significance	NA
*PDE6B* (NM_000283.4)	c.1920 + 1G > A	p.?	Pathogenic	NF	NA	4.19	NA	NA
	c.2470_2478del	p.(Lys824_Glu826del)	Uncertain significance	0.000128	NA	4.1599	NA	NA
*POC1B* (NM_172240.3)	c.1079_1080del	p.(Pro360Argfs*8)	Pathogenic	0.0000145	NA	5.69	NA	NA
*PROM1* (NM_006017.2)	exon 11 del	p.?	Pathogenic	NF	NA	NA	NA	NA
*PRPH2* (NM_000322.5)	c.440dup	p.(Gly148Trpfs*29)	Pathogenic	NF	NA	5.8699	NA	NA
	c.647C > A	p.(Pro216His)	Likely pathogenic	NF	12 of 13	5.0999	NA	NA
*RHO* (NM_000539.3)	c.670G > A	p.(Gly224Arg)	Likely pathogenic	0.0000159	12 of 13	5.0399	NA	NA
*RPGR* (NM_001 034853.2)	c.1366del	p.(Gln456Lysfs*20)	Pathogenic	NF	NA	4.73	NA	NA
*USH2A* (NM_206933.4)	c.4955C > T	p.(Pro1652Leu)	Likely pathogenic	0.000016	10 of 13	5.21	NA	NA
	c.6957 + 1G > C	p.?	Pathogenic	NF	NA	5.8099	NA	NA
	c.13894C > T	p.(Pro4632Ser)	Likely pathogenic	0.00000797	8 of 13	5.21	NA	NA

Column “Classification” refers to classification according to AMCG. ^*a*^Pathogenicity Scores from https://varsome.com/for missense variants (accessed November 2020) (BayesDel_addAF, DANN, DEOGEN2, EIGEN, FATHMM-MKL, LIST-S2, M-CAP, MVP, MutationAssessor, MutationTaster, PrimateAI, REVEL, and SIFT). Represent predictors supporting the pathogenic effect against the total of available predictors. ^*b*^GERP conservation score based on the reduction in the number of substitutions in the multi-species sequence alignment compared to the neutral expectation using the genomes of 35 mammals. Range: -12.3 to 6.17 (most conserved). ^*c*^HGMD public version (accessed November 2020). p.?, unknown protein effect; NF, no found; NA, not applicable. The “*” symbol corresponds to: stop-codon according to the format of mutations nomenclature from the Human Genome Variation Society (HGVS).

Copy number variants analysis with the bioinformatic tool allowed us to detect two putative deletions: a deletion in *PRPF31*, which was properly validated with MLPA (patient RPN-717), and a homozygous deletion of exon 11 of *PROM1* in patient RPN-709. In this last patient, the exon 11 did not have reads in the alignment and was not amplified by PCR; however, there was PCR amplification for the flanking exons, thus, reinforcing the hypothesis of a homozygous deletion of the exon.

The individual RPN-728 presented nephropathy and alterations in the brain MRI (mild hypoplasia of the cerebellar vermis and slightly elongated superior cerebellar peduncles) in addition to retinal degeneration, suggesting a Joubert syndrome. The panel sequencing allowed us to identify two pathogenic variants in *CEP290*, c.4966_4967delGA (p.Glu1656Asnfs^∗^3) and c.2817G > T (p.Lys939Asn). On the other hand, the panel analysis in the RPN-708 revealed a pathogenic variant in the *OTX2* gene (Table 1). Although this gene has been implicated in other diseases such as microphthalmia and retinal degeneration with pituitary dysfunction (OMIM: 610125; 610125), our patient only referred a macular dystrophy (MD) phenotype.

Because of the inclusion of the deep-intronic regions, we solved two cases who carried deep-intronic pathogenic variants in *ABCA4* and *CEP290* (RPN-750, RPN-734) (Table 1).

## Discussion

A total of 92 patients, previously clinically diagnosed with IRD, were analyzed by two IRD-custom panels. These gene panels allowed us to find a genetic diagnosis in 53 patients of 52 different families (Table 1), with a diagnostic ratio of 57.6%, which is within the average of other studies (50–70%) (Ellingford et al., 2016; Di Resta et al., 2018; Wang et al., 2018; Jespersgaard et al., 2019; Zenteno et al., 2020).

In recent years, several deep-intronic mutations have been described as pathogenic due to their effect in the splicing process by leading to the introduction of a pseudoexon (PE) in the coding sequence (Vaché et al., 2012; Braun et al., 2013; Zernant et al., 2014; Bauwens et al., 2015, 2019; Liquori et al., 2016; Baux et al., 2017; Fadaie et al., 2019; Khan et al., 2019; Sangermano et al., 2019). The inclusion of these regions in the present study allowed us to detect two deep intronic variants and, therefore, solve the molecular diagnosis in two more families. The variant *CEP290*: c.2991 + 1655A > G, detected in patient RPN-750, is one of the most prevalent in Leber congenital amaurosis (LCA) associated with *CEP290* (Den Hollander et al., 2006; Coppieters et al., 2010). Coppieters et al. reported this variant with a frequency of 49% in the *CEP290*-associated LCA cases. Also, we identified the deep-intronic variant c.4539 + 2064C > T in *ABCA4*. Deep-intronic variants in *ABCA4* were reported to be involved in 2.1–17.9% of the STGD cohorts (Braun et al., 2013; Zernant et al., 2014; Bauwens et al., 2015; Bax et al., 2015; Zaneveld et al., 2015; Schulz et al., 2017); our finding of 7.1% of cases within the STGD patients fits into the observed frequencies. Furthermore, the development of new therapeutical approaches, such as antisense oligonucleotides (AONs), entailed a new advantage for deep-intronic variants correction, which highlights the importance of detecting these changes for future treatments. In this sense, several studies have proven the therapeutic potential of AONs strategy and a phase I/II clinical trial for patients harboring the *CEP290* c.2991 + 1655A > G mutation using this type of therapy was conducted (Burke et al., 2012; Slijkerman et al., 2016; Duijkers et al., 2018; Cideciyan et al., 2019; Garanto et al., 2019; Sangermano et al., 2019; Xue and MacLaren, 2020).

In our study, *ABCA4* and *USH2A* are the most frequent mutated-genes, similar to other studies (Bernardis et al., 2016; Ezquerra-Inchausti et al., 2018; Jespersgaard et al., 2019). In *ABCA4*, 10% of alleles are reported to be complex (Shroyer et al., 2001; Zhang et al., 2015), a value that increased to 25% in our results. Moreover, we reported the variant c.3386G > T (p.Arg1129Leu) in *ABCA4* in 37.5% of the resolved patients with mutations in this gene (Table 1), which was not surprising because this variant appears to be very frequent in the Spanish population as we can observe in a study performed by Del Pozo-Valero et al. (2020). On the other hand, variants with an allelic frequency higher than 1% (MAF < 0.01) are usually ruled out as the cause of disease in prioritization analyses; however, in some cases, the frequency of some variants, although > 1%, is higher in patients than in healthy individuals, suggesting that they are causal for the disease. Examples of this have been reported for *ABCA4.* For instance, the variant c.5603A > T (p.Asn1868Ile) was presented in STGD patients four times more frequently than expected (Zernant et al., 2017), and it is a disease-causing variant in 5% of patients when it is in trans with a severe allele in *ABCA4*. This variant was initially considered benign, however, recent studies consider it pathogenic with reduced penetrance (Zernant et al., 2017; Cremers et al., 2018; Runhart et al., 2018). For autosomal dominant cases, *PRPH2* was found to be responsible for 14.8% of the resolved cases (Table 1), whilst a prevalence of 10.3% in *PRPH2* was the highest obtained thus far (Manes et al., 2015).

Concerning the patients who carry mutations in more than one IRD gene (Table 1), it is estimated that 2.7 billion individuals worldwide (36%) are carriers of an IRD disease-causing mutation, whereas 5.5 million are expected to be affected (Hanany et al., 2020). In this study, we diagnosed patient RPN-670, who was a carrier of disease-causing variants in *RP1*, *PRPH2*, and *USH2A* (Table 1). Despite the fact that any relative displays any symptoms, we cannot confirm that *PRPH2* and *RP1* variants are *de novo*, as DNA from family members was not available for segregation analysis. Incomplete penetrance can also be suggested, since it has been described for patients carrying pathogenic variants in *PRPH2* and *RP1* (Dietrich, 2002; Boon et al., 2008; Thiadens et al., 2012; Coco-Martin et al., 2020). It is important to remark that the fact of being a carrier of pathogenic variants in more than one gene can have a major impact on the reproductive choices for IRD patients, as well as impact their eligibility for gene-specific genetic therapies. This finding outstands the significance of achieving an exhaustive diagnosis.

The *OTX2* gene is associated with syndromic diseases, such as microphthalmia and retinal degeneration with or without pituitary dysfunction (OMIM: 610125; 610125). In this study, we also identified a nonsense variant in the *OTX2* gene in a family (FRPN-300) (Table 1) with an autosomal dominant mode of inheritance but only retinal symptoms. Similarly, two autosomal dominant families with heterozygous pathogenic variants in *OTX2* with only retinal degeneration patterns were previously reported (Vincent et al., 2014). Eleven truncating and two missense pathogenic variants have been described in this gene (LOVD accessed on April 7, 2021) and both type of variants have been associated with both syndromic and non-syndromic cases (Vincent et al., 2014; Slavotinek et al., 2015; Ellingford et al., 2016; Wang et al., 2016; Bryant et al., 2017; Patel et al., 2018; Sanchez-Navarro et al., 2018). Thus, a correlation between the type of mutation and the clinical diagnosis cannot be assessed.

A clear difference of the use of PV1 or PV2 concerning the solved cases (57.1% with the PV1 and 58.6% with PV2) did not exist. Strikingly, the rate of partially solved cases is almost half for the PV1 (11.1%) when compared to the PV2 (19.35%). It would be expected that the number of partially solved cases would be lower with a panel that includes all the deep-intronic mutations reported to date for *ABCA4* and *USH2A*. In our opinion, this could be due to the lower sample size analyzed with the PV2.

In general, custom panel designs remain an excellent option for the genetic diagnosis of IRD. However, due to the rapid increase of the number of genes involved, some groups prefer to use alternative approaches, such as WES (Lee et al., 2015; Riera et al., 2017; Zhang et al., 2018). Both sequencing strategies have their pros and cons. A specific panel of genes will allow the study of known IRD at a high time/effectiveness ratio regardless of the clinical diagnosis, which is an advantage in those cases in which the clinical diagnosis is not well defined or overlaps between different clinical entities. Furthermore, it allows not only the possibility of including a greater number of probes in repetitive regions such as ORF15 of the *RPGR* gene, which is highly involved in X-linked IRD cases (Vervoort et al., 2000; Megaw et al., 2015; Chiang et al., 2018; Charng et al., 2019), but also reported deep-intronic pathogenic variants (González-del Pozo et al., 2018; Di Scipio et al., 2020). On the other hand, WES made it possible to find mutations in novel IRD-related genes without updating the panel in low prevalent genes not included for the sake of increasing the depth of coverage or to identify novel IRD genes. The price reduction for high throughput sequencing has made the costs between the panels and WES quite even, so, it does not make the difference between the panels and WES. Taking this into account, the choice between both depends on the preferences of the clinician/researcher in terms of comprehensiveness, accuracy, and time consumption.

Currently, there is no significant difference between the panels and WES concerning the diagnostic rates, as the WES rate did not reach > 70% (Lee et al., 2015; Riera et al., 2017; Wang et al., 2018; Zhang et al., 2018; Liu et al., 2020). Therefore, further studies, including introns, other non-coding regions and epigenetics, are needed to achieve a comprehensive molecular diagnosis of IRD.

## Data Availability Statement

The data presented in this study are deposited in the European Genome-phenome Archive (EGA), Study: EGAS00001005369 and Dataset: EGAD00001007755.

## Ethics Statement

The studies involving human participants were reviewed and approved by the Hospital La Fe Ethics Committee. Written informed consent to participate in this study was provided by the participants’ legal guardian/next of kin.

## Author Contributions

EA, JM, and GG: conceptualization. BG and AR: methodology. BG, AR, EA, and TJ: resources. BG: writing—original draft preparation. BG, AR, EA, TJ, GG, and JM: writing—review and editing. GG and JM: supervision. JM: funding acquisition. All authors have read and agreed to the published version of the manuscript.

## Conflict of Interest

The authors declare that the research was conducted in the absence of any commercial or financial relationships that could be construed as a potential conflict of interest.

## Footnotes

1 https://sph.uth.edu/retnet/sum-dis.htm#A-genes

2 http://exac.broadinstitute.org/

3 https://gnomad.broadinstitute.org/

4 https://www.ncbi.nlm.nih.gov/clinvar/

5 https://grenada.lumc.nl/LSDB_list/lsdbs

6 https://portal.biobase-international.com/cgi-bin/portal/login.cgi

7 https://varsome.com/

8 http://www.umd.be/HSF

9 https://www.fruitfly.org/seq_tools/splice.html

10 https://mobidetails.iurc.montp.inserm.fr/MD/

11 http://software.broadinstitute.org/software/igv/

## References

[B1] Allikmets R. Singh N. Sun H. Shroyer N. F. Hutchinson A. Chidambaram A. (1997). A photoreceptor cell-specific ATP-binding transporter gene (ABCR) is mutated in recessive Starqardt macular dystrophy. *Nat. Genet.* 15 236–246. 10.1038/ng0397-236 9054934

[B2] Antiñolo G. Sánchez B. Borrego S. Rueda T. Chaparro P. Cabeza J. C. (1994). Identification of a new mutation at codon 171 of rhodopsin gene causing autosomal dominant retinitis pigmentosa. *Hum. Mol. Genet.* 3 1421–1421. 10.1093/hmg/3.8.1421 7987326

[B3] Ávila-Fernández A. Cantalapiedra D. Aller E. Vallespín E. Aguirre-Lambán J. Blanco-Kelly F. (2010). Mutation analysis of 272 Spanish families affected by autosomal recessive retinitis pigmentosa using a genotyping microarray. *Mol. Vis.* 16 2550–2558. 21151602 PMC3000238

[B4] Ayuso C. Millan J. M. (2010). Retinitis pigmentosa and allied conditions today: a paradigm of translational research. *Genome Med.* 2:34. 10.1186/gm155 20519033 PMC2887078

[B5] Bauwens M. De Zaeytijd J. Weisschuh N. Kohl S. Meire F. Dahan K. (2015). An augmented ABCA4 screen targeting noncoding regions reveals a deep intronic founder variant in belgian stargardt patients. *Hum. Mutat.* 36 39–42. 10.1002/humu.22716 25346251

[B6] Bauwens M. Garanto A. Sangermano R. Naessens S. Weisschuh N. De Zaeytijd J. (2019). ABCA4-associated disease as a model for missing heritability in autosomal recessive disorders: novel noncoding splice, cis-regulatory, structural, and recurrent hypomorphic variants. *Genet. Med.* 21 1761–1771. 10.1038/s41436-018-0420-y 30670881 PMC6752479

[B7] Baux D. Larrieu L. Blanchet C. Hamel C. Ben Salah S. Vielle A. (2007). Molecular and in silico analyses of the full-length isoform of usherin identify new pathogenic alleles in Usher type II patients. *Hum. Mutat.* 28 781–789. 10.1002/humu.20513 17405132

[B8] Baux D. Vaché C. Blanchet C. Willems M. Baudoin C. Moclyn M. (2017). Combined genetic approaches yield a 48% diagnostic rate in a large cohort of French hearing-impaired patients. *Sci. Rep.* 7:16783. 10.1038/s41598-017-16846-9 29196752 PMC5711943

[B9] Bax N. M. Sangermano R. Roosing S. Thiadens A. A. H. J. Hoefsloot L. H. van den Born L. I. (2015). Heterozygous deep-intronic variants and deletions in ABCA4 in persons with retinal dystrophies and one exonic ABCA4 variant. *Hum. Mutat.* 36 43–47. 10.1002/humu.22717 25363634

[B10] Bernardis I. Chiesi L. Tenedini E. Artuso L. Percesepe A. Artusi V. (2016). Unravelling the complexity of inherited retinal dystrophies molecular testing?: added value of targeted next-generation sequencing. *Biomed Res. Int.* 2016:6341870. 10.1155/2016/6341870 28127548 PMC5227126

[B11] Besnard T. García-García G. Baux D. Vaché C. Faugère V. Larrieu L. (2014). Experience of targeted Usher exome sequencing as a clinical test. *Mol. Genet. Genomic Med.* 2 30–43. 10.1002/mgg3.25 24498627 PMC3907913

[B12] Boon C. J. F. den Hollander A. I. Hoyng C. B. Cremers F. P. M. Klevering B. J. Keunen J. E. E. (2008). The spectrum of retinal dystrophies caused by mutations in the peripherin/RDS gene. *Prog. Retin. Eye Res.* 27 213–235. 10.1016/j.preteyeres.2008.01.002 18328765

[B13] Braun T. A. Mullins R. F. Wagner A. H. Andorf J. L. Johnston R. M. Bakall B. B. (2013). Non-exomic and synonymous variants in ABCA4 are an important cause of Stargardt disease. *Hum. Mol. Genet.* 22 5136–5145. 10.1093/hmg/ddt367 23918662 PMC3842174

[B14] Bravo-Gil N. Méndez-Vidal C. Romero-Pérez L. González-Del Pozo M. Rodríguez-De La Ruá E. Dopazo J. (2016). Improving the management of Inherited Retinal Dystrophies by targeted sequencing of a population-specific gene panel. *Sci. Rep.* 6:23910. 10.1038/srep23910 27032803 PMC4817143

[B15] Bryant L. Lozynska O. Maguire A. Aleman T. Bennett J. (2017). Prescreening whole exome sequencing results from patients with retinal degeneration for variants in genes associated with retinal degeneration. *Clin. Ophthalmol.* 12 49–63. 10.2147/OPTH.S147684 29343940 PMC5749571

[B16] Burke T. R. Fishman G. A. Zernant J. Schubert C. Tsang S. H. Theodore Smith R. (2012). Retinal phenotypes in patients homozygous for the G1961E mutation in the ABCA4 gene. *Invest. Ophthalmol. Vis. Sci.* 53 4458–4467. 10.1167/iovs.11-9166 22661473 PMC3394687

[B17] Carss K. Arno G. Erwood M. Stephens J. Sanchis-Juan A. Hull S. (2017). Comprehensive rare variant analysis via whole-genome sequencing to determine the molecular pathology of inherited retinal disease. *Am. J. Hum. Genet.* 100 75–90. 10.1016/j.ajhg.2016.12.003 28041643 PMC5223092

[B18] Charng J. Cideciyan A. V. Jacobson S. G. Sumaroka A. Schwartz S. B. Swider M. (2019). Variegated yet non-random rod and cone photoreceptor disease patterns in RPGR-ORF15-associated retinal degeneration. *Hum. Mol. Genet.* 28 175–175. 10.1093/hmg/ddy342 30285110 PMC6298234

[B19] Chiang J. P. W. Lamey T. M. Wang N. K. Duan J. Zhou W. McLaren T. L. (2018). Development of high-throughput clinical testing of RPGR ORF15 using a large inherited retinal dystrophy cohort. *Invest. Opthalmol. Vis. Sci.* 59:4434. 10.1167/iovs.18-24555 30193314

[B20] Cideciyan A. V. Jacobson S. G. Drack A. V. Ho A. C. Charng J. Garafalo A. V. (2019). Effect of an intravitreal antisense oligonucleotide on vision in Leber congenital amaurosis due to a photoreceptor cilium defect. *Nat. Med.* 25 225–228. 10.1038/s41591-018-0295-0 30559420

[B21] Coco-Martin R. M. Sanchez-Tocino H. T. Desco C. Usategui-Martín R. Tellería J. J. (2020). PRPH2-Related retinal diseases: broadening the clinical spectrum and describing a new mutation. *Genes (Basel)* 11:773. 10.3390/genes11070773 32660024 PMC7397286

[B22] Collin R. W. J. Littink K. W. Klevering B. J. van den Born L. I. Koenekoop R. K. Zonneveld M. N. (2008). Identification of a 2 Mb human ortholog of *Drosophila* eyes shut/spacemaker that is mutated in patients with retinitis pigmentosa. *Am. J. Hum. Genet.* 83 594–603. 10.1016/j.ajhg.2008.10.014 18976725 PMC2668042

[B23] Coppieters F. Casteels I. Meire F. De Jaegere S. Hooghe S. van Regemorter N. (2010). Genetic screening of LCA in Belgium: predominance of CEP290 and identification of potential modifier alleles in AHI1 of CEP290-related phenotypes. *Hum. Mutat.* 31 E1709–E1766. 10.1002/humu.21336 20683928 PMC3048164

[B24] Cornelis S. S. Bax N. M. Zernant J. Allikmets R. Fritsche L. G. den Dunnen J. T. (2017). In silico functional meta-analysis of 5,962 ABCA4 variants in 3,928 retinal dystrophy cases. *Hum. Mutat.* 38 400–408. 10.1002/humu.23165 28044389

[B25] Corton M. Tatu S. D. Avila-Fernandez A. Vallespín E. Tapias I. Cantalapiedra D. (2013). High frequency of CRB1 mutations as cause of Early-Onset Retinal Dystrophies in the Spanish population. *Orphanet J. Rare Dis.* 8:20. 10.1186/1750-1172-8-20 23379534 PMC3637806

[B26] Cremers F. (1998). Autosomal recessive retinitis pigmentosa and cone-rod dystrophy caused by splice site mutations in the Stargardt’s disease gene ABCR. *Hum. Mol. Genet.* 7 355–362. 10.1093/hmg/7.3.355 9466990

[B27] Cremers F. P. M. Cornelis S. S. Runhart E. H. Astuti G. D. N. (2018). Author response: penetrance of the ABCA4 p.Asn1868Ile allele in stargardt disease. *Invest. Opthalmol. Vis. Sci.* 59:5566. 10.1167/iovs.18-25944 30480704

[B28] Del Pozo-Valero M. Riveiro-Alvarez R. Blanco-Kelly F. Aguirre-Lamban J. Martin-Merida I. Iancu I.-F. (2020). Genotype–Phenotype correlations in a Spanish cohort of 506 families With biallelic ABCA4 pathogenic variants. *Am. J. Ophthalmol.* 219 195–204. 10.1016/j.ajo.2020.06.027 32619608

[B29] Den Hollander A. I. Koenekoop R. K. Yzer S. Lopez I. Arends M. L. Voesenek K. E. J. (2006). Mutations in the CEP290 (NPHP6) gene are a frequent cause of leber congenital amaurosis. *Am. J. Hum. Genet.* 79 556–561. 10.1086/507318 16909394 PMC1559533

[B30] den Hollander A. I. McGee T. L. Ziviello C. Banfi S. Dryja T. P. Gonzalez-Fernandez F. (2009). A homozygous missense mutation in the IRBP gene (RBP3) associated with autosomal recessive retinitis pigmentosa. *Invest. Ophthalmol. Vis. Sci.* 50 1864–1872. 10.1167/iovs.08-2497 19074801 PMC2823395

[B31] den Hollander A. I. ten Brink J. B. de Kok Y. J. M. van Soest S. van den Born L. I. van Driel M. A. (1999). Mutations in a human homologue of *Drosophila* crumbs cause retinitis pigmentosa (RP12). *Nat. Genet.* 23 217–221. 10.1038/13848 10508521

[B32] Di Resta C. Spiga I. Presi S. Merella S. Pipitone G. B. Manitto M. P. (2018). Integration of multigene panels for the diagnosis of hereditary retinal disorders using Next Generation Sequencing and bioinformatics approaches. *Electron. J. Int. Fed. Clin. Chem. Lab. Med.* 29 15–25. PMC5949615 29765283

[B33] Di Scipio M. Tavares E. Deshmukh S. Audo I. Green-Sanderson K. Zubak Y. (2020). Phenotype driven analysis of whole genome sequencing identifies deep intronic variants that cause retinal dystrophies by aberrant exonization. *Invest. Opthalmol. Vis. Sci.* 61:36. 10.1167/iovs.61.10.36 32881472 PMC7443117

[B34] Dietrich K. (2002). A novel mutation of the RP1 gene (Lys778ter) associated with autosomal dominant retinitis pigmentosa. *Br. J. Ophthalmol.* 86 328–332. 10.1136/bjo.86.3.328 11864893 PMC1771063

[B35] Diñeiro M. Capín R. Cifuentes G. Á Fernández−Vega B. Villota E. Otero A. (2020). Comprehensive genomic diagnosis of inherited retinal and optical nerve disorders reveals hidden syndromes and personalized therapeutic options. *Acta Ophthalmol.* 98 e1034–e1048. 10.1111/aos.14479 32483926 PMC7754416

[B36] Dryja T. P. Hahn L. B. Kajiwara K. Berson E. L. (1997). Dominant and digenic mutations in the peripherin/RDS and ROM1 genes in retinitis pigmentosa. *Invest. Ophthalmol. Vis. Sci.* 38 1972–1982. 9331261

[B37] Duijkers L. van den Born L. Neidhardt J. Bax N. Pierrache L. Klevering B. (2018). Antisense oligonucleotide-based splicing correction in individuals with leber congenital amaurosis due to compound heterozygosity for the c.2991+1655A&gt;G mutation in CEP290. *Int. J. Mol. Sci.* 19:753. 10.3390/ijms19030753 29518907 PMC5877614

[B38] Ellingford J. M. Barton S. Bhaskar S. O’Sullivan J. Williams S. G. Lamb J. A. (2016). Molecular findings from 537 individuals with inherited retinal disease. *J. Med. Genet.* 53 761–767. 10.1136/jmedgenet-2016-103837 27208204 PMC5106339

[B39] Ezquerra-Inchausti M. Anasagasti A. Barandika O. Garai-Aramburu G. Galdós M. López de Munain A. (2018). A new approach based on targeted pooled DNA sequencing identifies novel mutations in patients with Inherited Retinal Dystrophies. *Sci. Rep.* 8:15457. 10.1038/s41598-018-33810-3 30337596 PMC6194132

[B40] Fadaie Z. Khan M. Del Pozo-Valero M. Cornelis S. S. Ayuso C. Cremers F. P. M. (2019). Identification of splice defects due to noncanonical splice site or deep-intronic variants in ABCA4. *Hum. Mutat.* 40 2365–2376. 10.1002/humu.23890 31397521 PMC6899986

[B41] Farrar G. J. Carrigan M. Dockery A. Millington-Ward S. Palfi A. Chadderton N. (2017). Toward an elucidation of the molecular genetics of inherited retinal degenerations. *Hum. Mol. Genet.* 26 R2–R11. 10.1093/hmg/ddx185 28510639 PMC5886474

[B42] Fishman G. A. (2003). ABCA4 gene sequence variations in patients with autosomal recessive cone-rod dystrophy. *Arch. Ophthalmol.* 121:851. 10.1001/archopht.121.6.851 12796258

[B43] Fowler A. Mahamdallie S. Ruark E. Seal S. Ramsay E. Clarke M. (2016). Accurate clinical detection of exon copy number variants in a targeted NGS panel using DECoN. *Wellcome Open Res.* 1:20. 10.12688/wellcomeopenres.10069.1 28459104 PMC5409526

[B44] Garanto A. Duijkers L. Tomkiewicz T. Z. Collin R. W. J. (2019). Antisense oligonucleotide screening to optimize the rescue of the splicing defect caused by the recurrent deep-intronic ABCA4 Variant c.4539+2001G&gt;A in stargardt disease. *Genes (Basel)* 10:452. 10.3390/genes10060452 31197102 PMC6628380

[B45] Gerber S. Rozet J. M. van de Pol T. J. R. Hoyng C. B. Munnich A. Blankenagel A. (1998). Complete exon–intron structure of the retina-specific ATP binding transporter gene (ABCR) allows the identification of novel mutations underlying stargardt disease. *Genomics* 48 139–142. 10.1006/geno.1997.5164 9503029

[B46] Glockle N. Kohl S. Mohr J. Scheurenbrand T. Sprecher A. Weisschuh N. (2014). Panel-based next generation sequencing as a reliable and efficient technique to detect mutations in unselected patients with retinal dystrophies. *Eur. J. Hum. Genet.* 22 99–104. 10.1038/ejhg.2013.72 23591405 PMC3865404

[B47] González-del Pozo M. Borrego S. Barragán I. Pieras J. I. Santoyo J. Matamala N. (2011). Mutation screening of multiple genes in Spanish patients with autosomal recessive retinitis pigmentosa by targeted resequencing. *PLoS One* 6:27894. 10.1371/journal.pone.0027894 22164218 PMC3229495

[B48] González-del Pozo M. Martín-Sánchez M. Bravo-Gil N. Méndez-Vidal C. Chimenea Á Rodríguez-de la Rúa E. (2018). Searching the second hit in patients with inherited retinal dystrophies and monoallelic variants in ABCA4, USH2A and CEP290 by whole-gene targeted sequencing. *Sci. Rep.* 8:13312. 10.1038/s41598-018-31511-5 30190494 PMC6127285

[B49] Gonzalez-Fernandez F. Kurz D. Bao Y. Newman S. Conway B. P. Young J. E. (1999). 11-cis retinol dehydrogenase mutations as a major cause of the congenital night-blindness disorder known as fundus albipunctatus. *Mol. Vis.* 5:41. 10617778

[B50] Hanany M. Rivolta C. Sharon D. (2020). Worldwide carrier frequency and genetic prevalence of autosomal recessive inherited retinal diseases. *Proc. Natl. Acad. Sci. U.S.A.* 117 2710–2716. 10.1073/pnas.1913179117 31964843 PMC7007541

[B51] Hu F. Gao F. Li J. Xu P. Wang D. Chen F. (2020). Novel variants associated with Stargardt disease in Chinese patients. *Gene* 754:144890. 10.1016/j.gene.2020.144890 32534057

[B52] Jacobson S. G. Cideciyan A. V. Iannaccone A. Weleber R. G. Fishman G. A. Maguire A. M. (2000). Disease expression of RP1 mutations causing autosomal dominant retinitis pigmentosa. *Invest. Ophthalmol. Vis. Sci.* 41 1898–1908. 10845615

[B53] Jamshidi F. Place E. M. Mehrotra S. Navarro-Gomez D. Maher M. Branham K. E. (2019). Contribution of noncoding pathogenic variants to RPGRIP1-mediated inherited retinal degeneration. *Genet. Med.* 21 694–704. 10.1038/s41436-018-0104-7 30072743 PMC6399075

[B54] Jespersgaard C. Fang M. Bertelsen M. Dang X. Jensen H. Chen Y. (2019). Molecular genetic analysis using targeted NGS analysis of 677 individuals with retinal dystrophy. *Sci. Rep.* 9:1219. 10.1038/s41598-018-38007-2 30718709 PMC6362094

[B55] Kajiwara K. Berson E. Dryja T. (1994). Digenic retinitis pigmentosa due to mutations at the unlinked peripherin/RDS and ROM1 loci. *Science* 264 1604–1608. 10.1126/science.8202715 8202715

[B56] Khan M. Cornelis S. S. Khan M. I. Elmelik D. Manders E. Bakker S. (2019). Cost-effective molecular inversion probe-based ABCA4 sequencing reveals deep-intronic variants in Stargardt disease. *Hum. Mutat.* 40 1749–1759. 10.1002/humu.23787 31212395

[B57] Kinnick T. R. Mullins R. F. Dev S. Leys M. Mackey D. A. Kay C. N. (2011). Autosomal recessive vitelliform macular dystrophy in a large cohort of vitelliform macular dystrophy patients. *Retina* 31 581–595. 10.1097/IAE.0b013e318203ee60 21273940

[B58] Kohl S. Christ-Adler M. Apfelstedt-Sylla E. Kellner U. Eckstein A. Zrenner E. (1997). RDS/peripherin gene mutations are frequent causes of central retinal dystrophies. *J. Med. Genet.* 34 620–626. 10.1136/jmg.34.8.620 9279751 PMC1051021

[B59] Koyanagi Y. Akiyama M. Nishiguchi K. M. Momozawa Y. Kamatani Y. Takata S. (2019). Genetic characteristics of retinitis pigmentosa in 1204 Japanese patients. *J. Med. Genet.* 56 662–670. 10.1136/jmedgenet-2018-105691 31213501

[B60] Le Quesne Stabej P. Saihan Z. Rangesh N. Steele-Stallard H. B. Ambrose J. Coffey A. (2012). Comprehensive sequence analysis of nine Usher syndrome genes in the UK National Collaborative Usher Study. *J. Med. Genet.* 49 27–36. 10.1136/jmedgenet-2011-100468 22135276 PMC3678402

[B61] Lee K. Berg J. S. Milko L. Crooks K. Lu M. Bizon C. (2015). High diagnostic yield of whole exome sequencing in participants with retinal dystrophies in a clinical ophthalmology setting. *Am. J. Ophthalmol.* 160 354–363.e9. 10.1016/j.ajo.2015.04.026 25910913 PMC4506879

[B62] Lenassi E. Vincent A. Li Z. Saihan Z. Coffey A. J. Steele-Stallard H. B. (2015). A detailed clinical and molecular survey of subjects with nonsyndromic USH2A retinopathy reveals an allelic hierarchy of disease-causing variants. *Eur. J. Hum. Genet.* 23 1318–1327. 10.1038/ejhg.2014.283 25649381 PMC4592079

[B63] Lewis R. A. Shroyer N. F. Singh N. Allikmets R. Hutchinson A. Li Y. (1999). Genotype/Phenotype analysis of a photoreceptor-specific ATP-Binding cassette transporter gene, ABCR, in stargardt disease. *Am. J. Hum. Genet.* 64 422–434. 10.1086/302251 9973280 PMC1377752

[B64] Liquori A. Vaché C. Baux D. Blanchet C. Hamel C. Malcolm S. (2016). Whole USH2A gene sequencing identifies several new deep intronic mutations. *Hum. Mutat.* 37 184–193. 10.1002/humu.22926 26629787

[B65] Littink K. W. Pott J.-W. R. Collin R. W. J. Kroes H. Y. Verheij J. B. G. M. Blokland E. A. W. (2010). A novel nonsense mutation in CEP290 induces exon skipping and leads to a relatively mild retinal phenotype. *Invest. Opthalmol. Vis. Sci.* 51 3646. 10.1167/iovs.09-5074 20130272

[B66] Liu X. Z. Li Y. Y. Yang L. P. (2020). [Comparison study of whole exome sequencing and targeted panel sequencing in molecular diagnosis of inherited retinal dystrophies]. *Beijing Da Xue Xue Bao* 52 836–844. 33047716 10.19723/j.issn.1671-167X.2020.05.007 PMC7653409

[B67] Lotery A. J. (2001). Mutations in the CRB1 gene cause leber congenital amaurosis. *Arch. Ophthalmol.* 119:415. 10.1001/archopht.119.3.415 11231775

[B68] Manes G. Guillaumie T. Vos W. L. Devos A. Zeitz C. Marquette V. (2015) High prevalence of PRPH2 in autosomal dominant retinitis pigmentosa in france and characterization of biochemical and clinical features. *Am. J. Ophthalmol.* 159 302–314. 10.1016/j.ajo.2014.10.033 25447119

[B69] Maw M. Kumaramanickavel G. Kar B. John S. Bridges R. Denton M. (1998). Two Indian siblings with Oguchi disease are homozygous for an arrestin mutation encoding premature termination. *Hum. Mutat.* 11 S317–S319. 10.1002/humu.1380110199 9452120

[B70] Megaw R. D. Soares D. C. Wright A. F. (2015). RPGR: its role in photoreceptor physiology, human disease, and future therapies. *Exp. Eye Res.* 138 32–41. 10.1016/j.exer.2015.06.007 26093275 PMC4553903

[B71] Nasonkin I. Illing M. Koehler M. R. Schmid M. Molday R. S. Weber B. H. F. (1998). Mapping of the rod photoreceptor ABC transporter (ABCR) to 1p21-p22.1 and identification of novel mutations in Stargardt’s disease. *Hum. Genet.* 102 21–26. 10.1007/s004390050649 9490294

[B72] Neveling K. den Hollander A. I. Cremers F. P. M. Collin R. W. J. (2012). “Identification and analysis of inherited retinal disease genes,” in *Retinal Degeneration. Methods in Molecular Biology (Methods and Protocols)*, Vol. 935 eds Weber B. LANGMANN T. (Totowa, NJ: Humana Press), 3–23. 10.1007/978-1-62703-080-9_1 23150357

[B73] Nishimura D. Y. Baye L. M. Perveen R. Searby C. C. Avila-Fernandez A. Pereiro I. (2010). Discovery and functional analysis of a retinitis pigmentosa gene, C2ORF71. *Am. J. Hum. Genet.* 86 686–695. 10.1016/j.ajhg.2010.03.005 20398886 PMC2868997

[B74] Parmeggiani F. Sorrentino F. S. Ponzin D. Barbaro V. Ferrari S. Di Iorio E. (2011). Retinitis pigmentosa: genes and disease mechanisms. *Curr. Genomics* 12 238–249. 10.2174/138920211795860107 22131869 PMC3131731

[B75] Patel N. Khan A. O. Alsahli S. Abdel-Salam G. Nowilaty S. R. Mansour A. M. (2018). Genetic investigation of 93 families with microphthalmia or posterior microphthalmos. *Clin. Genet.* 93 1210–1222. 10.1111/cge.13239 29450879

[B76] Payne A. M. Downes S. M. Bessant D. A. R. Bird A. C. Bhattacharya S. S. (1998). Founder effect, seen in the british population, of the 172 Peripherin/RDS mutation—and further refinement of genetic positioning of the Peripherin/RDS gene. *Am. J. Hum. Genet.* 62 192–195. 10.1086/301679 9443872 PMC1376804

[B77] Perea-Romero I. Gordo G. Iancu I. F. Del Pozo-Valero M. Almoguera B. Blanco-Kelly F. (2021). Genetic landscape of 6089 inherited retinal dystrophies affected cases in Spain and their therapeutic and extended epidemiological implications. *Sci. Rep.* 11:1526. 10.1038/s41598-021-81093-y 33452396 PMC7810997

[B78] Richards S. Aziz N. Bale S. Bick D. Das S. Gastier-Foster J. (2015). Standards and guidelines for the interpretation of sequence variants: a joint consensus recommendation of the American College of Medical Genetics and Genomics and the Association for Molecular Pathology. *Genet. Med.* 17 405–423. 10.1038/gim.2015.30 25741868 PMC4544753

[B79] Riera M. Navarro R. Ruiz-Nogales S. Méndez P. Burés-Jelstrup A. Corcóstegui B. (2017). Whole exome sequencing using Ion Proton system enables reliable genetic diagnosis of inherited retinal dystrophies. *Sci. Rep.* 7:42078. 10.1038/srep42078 28181551 PMC5299602

[B80] Rivolta C. Berson E. L. Dryja T. P. (2002). Paternal uniparental heterodisomy with partial isodisomy of chromosome 1 in a patient with retinitis pigmentosa without hearing loss and a missense mutation in the Usher syndrome type II gene USH2A. *Arch. Ophthalmol.* 120 1566–1571. 12427073 10.1001/archopht.120.11.1566

[B81] Rivolta C. Sweklo E. A. Berson E. L. Dryja T. P. (2000). Report missense mutation in the USH2A gene: association with recessive retinitis pigmentosa without hearing loss. *Am. J. Hum. Genet* 66 1975–1978. 10.1086/302926 10775529 PMC1378039

[B82] Rodríguez-Muñoz A. Aller E. Jaijo T. González-García E. Cabrera-Peset A. Gallego-Pinazo R. (2020). Expanding the clinical and molecular heterogeneity of nonsyndromic inherited retinal dystrophies. *J. Mol. Diagnostics* 22 532–543. 10.1016/j.jmoldx.2020.01.003 32036094

[B83] Rozet J. M. Gerber S. Souied E. Perrault I. Châtelin S. Ghazi I. (1998). Spectrum of ABCR gene mutations in autosomal recessive macular dystrophies. *Eur. J. Hum. Genet.* 6 291–295. 10.1038/sj.ejhg.5200221 9781034

[B84] Runhart E. H. Sangermano R. Cornelis S. S. Verheij J. B. G. M. Plomp A. S. Boon C. J. F. (2018). The common ABCA4 Variant p.Asn1868Ile shows nonpenetrance and variable expression of stargardt disease when present in trans with severe variants. *Invest. Opthalmol. Vis. Sci.* 59 3220. 10.1167/iovs.18-23881 29971439

[B85] Sahel J. A. Marazova K. Audo I. (2015). Clinical characteristics and current therapies for inherited retinal degenerations. *Cold Spring Harb. Perspect. Med.* 5 1–25. 10.1101/cshperspect.a017111 25324231 PMC4315917

[B86] Salles M. V. Motta F. L. Martin R. Filippelli-Silva R. Dias da Silva E. Varela P. (2018). Variants in the ABCA4 gene in a Brazilian population with Stargardt disease. *Mol. Vis.* 24 546–559. 30093795 PMC6070459

[B87] Salmaninejad A. Motaee J. Farjami M. Alimardani M. Esmaeilie A. Pasdar A. (2019). Next-generation sequencing and its application in diagnosis of retinitis pigmentosa. *Ophthalmic Genet.* 40 393–402. 10.1080/13816810.2019.1675178 31755340

[B88] Sanchez-Navarro R. J. I da Silva L. Blanco-Kelly F. Zurita O. Sanchez-Bolivar N. Villaverde C. (2018). Combining targeted panel-based resequencing and copy-number variation analysis for the diagnosis of inherited syndromic retinopathies and associated ciliopathies. *Sci. Rep.* 8:5285. 10.1038/s41598-018-23520-1 29588463 PMC5869593

[B89] Sanchis-Juan A. Stephens J. French C. Gleadall N. Mégy K. Penkett C. (2018). Complex structural variants resolved by short-read and long-read whole genome sequencing in mendelian disorders. *BioRxiv* [preprint] 10.1101/281683 281683 PMC6286558 30526634

[B90] Sangermano R. Garanto A. Khan M. Runhart E. H. Bauwens M. Bax N. M. (2019). Deep-intronic ABCA4 variants explain missing heritability in Stargardt disease and allow correction of splice defects by antisense oligonucleotides. *Genet. Med.* 21 1751–1760. 10.1038/s41436-018-0414-9 30643219 PMC6752325

[B91] Schulz H. L. Grassmann F. Kellner U. Spital G. Rüther K. Jägle H. (2017). Mutation spectrum of the ABCA4 gene in 335 stargardt disease patients from a multicenter german cohort—impact of selected deep intronic variants and common SNPs. *Invest. Opthalmol. Vis. Sci.* 58:394. 10.1167/iovs.16-19936 28118664 PMC5270621

[B92] Schwartz S. B. Aleman T. S. Cideciyan A. V. Windsor E. A. M. Sumaroka A. Roman A. J. (2005). Disease expression in usher syndrome caused by VLGR1 gene mutation (USH2C) and comparison with USH2A phenotype. *Invest. Opthalmol. Vis. Sci.* 46:734. 10.1167/iovs.04-1136 15671307

[B93] Sheck L. Davies W. I. L. Moradi P. Robson A. G. Kumaran N. Liasis A. C. (2018). Leber congenital amaurosis associated with mutations in CEP290, clinical phenotype, and natural history in preparation for trials of novel therapies. *Ophthalmology* 125 894–903. 10.1016/j.ophtha.2017.12.013 29398085 PMC5974693

[B94] Shroyer N. F. Lewis R. A. Yatsenko A. N. Lupski J. R. (2001). Null missense ABCR (ABCA4) mutations in a family with stargardt disease and retinitis pigmentosa. *Invest. Ophthalmol. Vis. Sci.* 42 2757–2761. 11687513

[B95] Slavotinek A. M. Garcia S. T. Chandratillake G. Bardakjian T. Ullah E. Wu D. (2015). Exome sequencing in 32 patients with anophthalmia/microphthalmia and developmental eye defects. *Clin. Genet.* 88 468–473. 10.1111/cge.12543 25457163 PMC4452457

[B96] Slijkerman R. W. Vaché C. Dona M. García-García G. Claustres M. Hetterschijt L. (2016). Antisense oligonucleotide-based splice correction for USH2A-associated Retinal degeneration caused by a frequent deep-intronic mutation. *Mol. Ther. Nucleic Acids* 5:e381. 10.1038/mtna.2016.89 27802265

[B97] Srivastava S. Ramsbottom S. A. Molinari E. Alkanderi S. Filby A. White K. (2017). A human patient-derived cellular model of Joubert syndrome reveals ciliary defects which can be rescued with targeted therapies. *Hum. Mol. Genet.* 26 4657–4667. 10.1093/hmg/ddx347 28973549 PMC5886250

[B98] Stenirri S. Fermo I. Battistella S. Galbiati S. Soriani N. Paroni R. (2004). Denaturing HPLC profiling of the ABCA4 gene for reliable detection of allelic variations. *Clin. Chem.* 50 1336–1343. 10.1373/clinchem.2004.033241 15192030

[B99] Stone E. M. Andorf J. L. Whitmore S. S. DeLuca A. P. Giacalone J. C. Streb L. M. (2017). Clinically focused molecular investigation of 1000 consecutive families with inherited retinal disease. *Ophthalmology* 124 1314–1331. 10.1016/j.ophtha.2017.04.008 28559085 PMC5565704

[B100] Tatour Y. Ben-Yosef T. (2020). Syndromic inherited retinal diseases: genetic, clinical and diagnostic aspects. *Diagnostics* 10:779. 10.3390/diagnostics10100779 33023209 PMC7600643

[B101] Thiadens A. A. H. J. Phan T. M. L. Zekveld-Vroon R. C. Leroy B. P. Van Den Born L. I. Hoyng C. B. (2012). Clinical course, genetic etiology, and visual outcome in cone and cone-rod dystrophy. *Ophthalmology* 119 819–826. 10.1016/j.ophtha.2011.10.011 22264887

[B102] To K. Adamian M. Berson E. L. (2004). Histologic study of retinitis pigmentosa due to a mutation in the RP13 gene (PRPC8): comparison with rhodopsin Pro23His, Cys110Arg, and Glu181Lys. *Am. J. Ophthalmol.* 137 946–948. 10.1016/j.ajo.2003.10.047 15126168

[B103] Trujillo M. J. Martinez-Gimeno M. Gimenez A. Lorda I. Bueno J. Garcia-Sandoval B. (2001). Two novel mutations (Y141H; C214Y) and previously published mutation (R142W) in the RDS-peripherin gene in autosomal dominant macular dystrophies in Spanish families. *Hum. Mutat.* 17:80. 10.1002/1098-1004(2001)17:1<80::AID-HUMU27>3.0.CO;2-G 11139263

[B104] Vaché C. Besnard T. le Berre P. García-García G. Baux D. Larrieu L. (2012). Usher syndrome type 2 caused by activation of an USH2A pseudoexon: implications for diagnosis and therapy. *Hum. Mutat.* 33 104–108. 10.1002/humu.21634 22009552

[B105] Vervoort R. Lennon A. Bird A. C. Tulloch B. Axton R. Miano M. G. (2000). Mutational hot spot within a new RPGR exon in X-linked retinitis pigmentosa. *Nat. Genet.* 25 462–466. 10.1038/78182 10932196

[B106] Vincent A. Forster N. Maynes J. T. Paton T. A. Billingsley G. Roslin N. M. (2014). OTX2 mutations cause autosomal dominant pattern dystrophy of the retinal pigment epithelium. *J. Med. Genet.* 51 797–805. 10.1136/jmedgenet-2014-102620 25293953

[B107] Wang L. Zhang J. Chen N. Wang L. Zhang F. Ma Z. (2018). Application of whole exome and targeted panel sequencing in the clinical molecular diagnosis of 319 Chinese families with inherited retinal dystrophy and comparison study. *Genes (Basel)* 9 1–11. 10.3390/genes9070360 30029497 PMC6071067

[B108] Wang S. Zhang Q. Zhang X. Wang Z. Zhao P. (2016). Clinical and genetic characteristics of Leber congenital amaurosis with novel mutations in known genes based on a Chinese eastern coast Han population. *Graefes Arch. Clin. Exp. Ophthalmol.* 254 2227–2238. 10.1007/s00417-016-3428-5 27422788

[B109] Webb T. R. Parfitt D. A. Gardner J. C. Martinez A. Bevilacqua D. Davidson A. E. (2012). Deep intronic mutation in OFD1, identified by targeted genomic next-generation sequencing, causes a severe form of X-linked retinitis pigmentosa (RP23). *Hum. Mol. Genet.* 21 3647–3654. 10.1093/hmg/dds194 22619378 PMC3406759

[B110] Xue K. MacLaren R. E. (2020). Antisense oligonucleotide therapeutics in clinical trials for the treatment of inherited retinal diseases. *Expert Opin. Invest. Drugs* 29 1163–1170. 10.1080/13543784.2020.1804853 32741234

[B111] Zaneveld J. Siddiqui S. Li H. Wang X. Wang H. Wang K. (2015). Comprehensive analysis of patients with Stargardt macular dystrophy reveals new genotype–phenotype correlations and unexpected diagnostic revisions. *Genet. Med.* 17 262–270. 10.1038/gim.2014.174 25474345 PMC4385427

[B112] Zenteno J. C. García-Montaño L. A. Cruz-Aguilar M. Ronquillo J. Rodas-Serrano A. Aguilar-Castul L. (2020). Extensive genic and allelic heterogeneity underlying inherited retinal dystrophies in Mexican patients molecularly analyzed by next-generation sequencing. *Mol. Genet. Genomic Med.* 8 1–17. 10.1002/mgg3.1044 31736247 PMC6978239

[B113] Zernant J. Lee W. Collison F. T. Fishman G. A. Sergeev Y. V. Schuerch K. (2017). Frequent hypomorphic alleles account for a significant fraction of ABCA4 disease and distinguish it from age-related macular degeneration. *J. Med. Genet.* 54 404–412. 10.1136/jmedgenet-2017-104540 28446513 PMC5786429

[B114] Zernant J. Schubert C. Im K. M. Burke T. Brown C. M. Fishman G. A. (2011). Analysis of the ABCA4 gene by next-generation sequencing. *Invest. Ophthalmol. Vis. Sci.* 52 8479–8487. 10.1167/iovs.11-8182 21911583 PMC3208189

[B115] Zernant J. Xie Y. A. Ayuso C. Riveiro-Alvarez R. Lopez-Martinez M.-A. Simonelli F. (2014). Analysis of the ABCA4 genomic locus in Stargardt disease. *Hum. Mol. Genet.* 23 6797–6806. 10.1093/hmg/ddu396 25082829 PMC4245042

[B116] Zhang L. Sun Z. Zhao P. Huang L. Xu M. Yang Y. (2018). Whole-exome sequencing revealed HKDC1 as a candidate gene associated with autosomal-recessive retinitis pigmentosa. *Hum. Mol. Genet.* 27 4157–4168. 10.1093/hmg/ddy281 30085091 PMC6240732

[B117] Zhang N. Tsybovsky Y. Kolesnikov A. V. Rozanowska M. Swider M. Schwartz S. B. (2015). Protein misfolding and the pathogenesis of ABCA4-associated retinal degenerations. *Hum. Mol. Genet.* 24 3220–3237. 10.1093/hmg/ddv073 25712131 PMC4424957

