# Supplementary material for: Updating the Genetic Landscape of Inherited Retinal Dystrophies
Source: Front Cell Dev Biol. 2021 Jul 13;9:645600. doi: 10.3389/fcell.2021.645600 (PMC8315279; doi:10.3389/fcell.2021.645600)
Supplement: Supplementary file 1 [file Data_Sheet_1.docx]

| **Table S1. Genes included in PV1.** | | | | | | | | | |
| --- | --- | --- | --- | --- | --- | --- | --- | --- | --- |
| *ABCA4* | *CA4* | *CNGB3* | *GDF6* | *KIZ* | | *OTX2* | *PRPF6* | *RHO* | *SLC7A14* |
| *ADAM9* | *CABP4* | *CNNM4* | *GNAT2* | *KLHL7* | | *PDE6A* | *PRPF8* | *RIMS1* | *SNRNP200* |
| *ADAMTS18* | *CACNA1F* | *CRB1* | *GUCA1A* | *LCA5* | | *PDE6B* | *PRPH2* | *RLBP1* | *SPATA7* |
| *AIPL1* | *CACNA2D4* | *CRX* | *GUCA1B* | *LRAT* | | *PDE6C* | *RAB28* | *ROM1* | *TIMP3* |
| *ARL2BP* | *CDH3* | *DHDDS* | *GUCY2D* | *MAK* | | *PDE6G* | *RAX2* | *RP1* | *TOPORS* |
| *ARL6* | *CDHR1* | *DRAM2* | *HK1* | *MERTK* | | *PDE6H* | *RBP3* | *RP1L1* | *TTC8* |
| *BBS1* | *CEP290* | *DTHD1* | *IDH3B* | *MVK* | | *PITPNM3* | *RBP4* | *RP2* | *TTLL5* |
| *BBS2* | *CERKL* | *EFEMP1* | *IMPDH1* | *NEK2* | | *POC1B* | *RD3* | *RP9* | *TULP1* |
| *BEST1* | *CHM* | *ELOVL4* | *IMPG1* | *NEUROD1* | | *PRCD* | *RDH12* | *RPE65* | *UNC119* |
| *C1QTNF5* | *CLRN1* | *EYS* | *IMPG2* | *NMNAT1* | | *PROM1* | *RDH5* | *RPGR* | *USH1C* |
| *C21orf2* | *CNGA1* | *FAM161A* | *IQCB1* | *NR2E3* | | *PRPF3* | *RGR* | *RPGRIP1* | *USH2A* |
| *PCARE* | *CNGA3* | *FLVCR1* | *KCNJ13* | *NRL* | | *PRPF31* | *RGS9* | *SAG* | *ZNF408* |
| *C8orf37* | *CNGB1* | *FSCN2* | *KCNV2* | *OFD1* | | *PRPF4* | *RGS9BP* | *SEMA4A* | *ZNF513* |
| chr1:216064520-216064560 (*USH2A*) | | | chr1:94492980-94493020 (*ABCA4*) | | | | chr12:88494940-88494980 (*CEP290*) | | |
| chr19:54633379-54633419 (*PRPF31*) | | | | | chrX:13770172-13770212 (*ODF1*) | | | | |

Genomic hg19 coordinates correspond to deep-intronic regions in which variants have been previously described as

pathogenic.

| **Table S2.** Genes included in PV2. | | | | | | |
| --- | --- | --- | --- | --- | --- | --- |
| *ABCA4* | *CDHR1* | *GNAT2* | *MAK* | *PRPF31* | *RP1L1* | chr1:216064520-216064560 (*USH2A*) |
| *ADAM9* | *CEP290* | *GUCA1A* | *MERTK* | *PRPF4* | *RP2* | chr1:215967733-215967833 (*USH2A*) |
| *ADAMTS18* | *CERKL* | *GUCA1B* | *NEK2* | *PRPF6* | *RPE65* | chr1:215827262-215827362 (*USH2A*) |
| *AIPL1* | *CHM* | *GUCY2D* | *MFSD8* | *PRPF8* | *RPGR* | chr1:94492980-94493020 (*ABCA4*) |
| *ARL2BP* | *CNGA1* | *HK1* | *NMNAT1* | *PRPH2* | *RPGRIP1* | chr1:94549552-94549652 (*ABCA4*) |
| *ARL3* | *CNGA3* | *IDH3B* | *NR2E3* | *RAB28* | *SAMD11* | chr1:94549731-94549831 (*ABCA4*) |
| *ARL6* | *CNGB1* | *IFT140* | *NRL* | *RAX2* | *SAG* | chr1:94526884-94526984 (*ABCA4*) |
| *BBS1* | *CNGB3* | *IFT172* | *OFD1* | *RBP3* | *SEMA4A* | chr1:94546730-94546864 (*ABCA4*) |
| *BBS2* | *CNNM4* | *IMPDH1* | *OTX2* | *RBP4* | *SLC7A14* | chr1:94527648-94527748 (*ABCA4*) |
| *BEST1* | *CRB1* | *IMPG1* | *PDE6A* | *RD3* | *SNRNP200* | chr1:94511076-94511176 (*ABCA4*) |
| *C1QTNF5* | *CRX* | *IMPG2* | *PDE6B* | *RDH12* | *SPATA7* | chr1:94509749-94509849 (*ABCA4*) |
| *C21orf2* | *CTNNA1* | *IQCB1* | *PDE6C* | *RDH5* | *TOPORS* | chr1:94496459-94496559 (*ABCA4*) |
| *PCARE* | *DHDDS* | *KCNJ13* | *PDE6G* | *REEP6* | *TTC8* | chr1:94494092-94494192 (*ABCA4*) |
| *C8orf37* | *DRAM2* | *KCNV2* | *PDE6H* | *RGR* | *TTLL5* | chr1:94493851-94493951 (*ABCA4*) |
| *CA4* | *ELOVL4* | *KIAA1549* | *PITPNM3* | *RHO* | *TULP1* | chr1:94492886-94493050 (*ABCA4*) |
| *CABP4* | *EYS* | *KIZ* | *POC1B* | *RIMS1* | *UNC119* | chr1:94484032-94484132 (*ABCA4*) |
| *CACNA1F* | *FAM161A* | *KLHL7* | *PRCD* | *RLBP1* | *USH2A* | chr1:94483872-94484052 (*ABCA4*) |
| *CACNA2D4* | *PRDM3* | *LCA5* | *PROM1* | *ROM1* | *ZNF408* | chr1:94481917-94482017 (*ABCA4*) |
| *CDH3* | *GDF6* | *LRAT* | *PRPF3* | *RP1* | *ZNF513* | chr12:88494940-88494980 (*CEP290*) |
| chr1:216039671-216039771 (*USH2A*) | | | | | | chr19:54633379-54633419 (*PRPF31*) |
| chr1:216247426-216247526 (*USH2A*) | | | | | | chrX:13770172-13770212 (*ODF1*) |

Genomic hg19 coordinates correspond to deep-intronic regions already present in PV1 and those in which new variants

have been described as pathogenic post-PV1.

| **Table S3.** Deep-intronic variants described in *USH2A* and *ABCA4* as pathogenic. | | |
| --- | --- | --- |
| **Gene** | **Nucleotide change** | **Reference** |
| *USH2A*  (NM_206933.2) | c.7595-2144A>G | (15) |
|  | c.5573-843A>G | (16) |
|  | c.8845+628C>T |  |
|  | c.9959-4159A>G |  |
|  | c.14134-3169A>G | (17) |
| *ABCA4*  (NM_000350.2) | c.5196+1056A>G | (14) |
|  | c.5196+1216C>A |  |
|  | c.1938−619A>G | (18) |
|  | c.4539+2064C>T |  |
|  | c.4539+2001G>A | (19) |
|  | c.769-605C>T | (20) |
|  | c.4539+859C>T |  |
|  | c.4539+2065C>G |  |
|  | c.2919−826T>A | (21) |
|  | c.3050+370C>T |  |
|  | c.769–784T>C | (22) |
|  | c.4253+43G>A |  |
|  | c.859-506G>C |  |
|  | c.1937+435C>G |  |
|  | c.4539+1100A>G |  |
|  | c.859-540C>G | (23) |
|  | c.5197-557G>T |  |

**
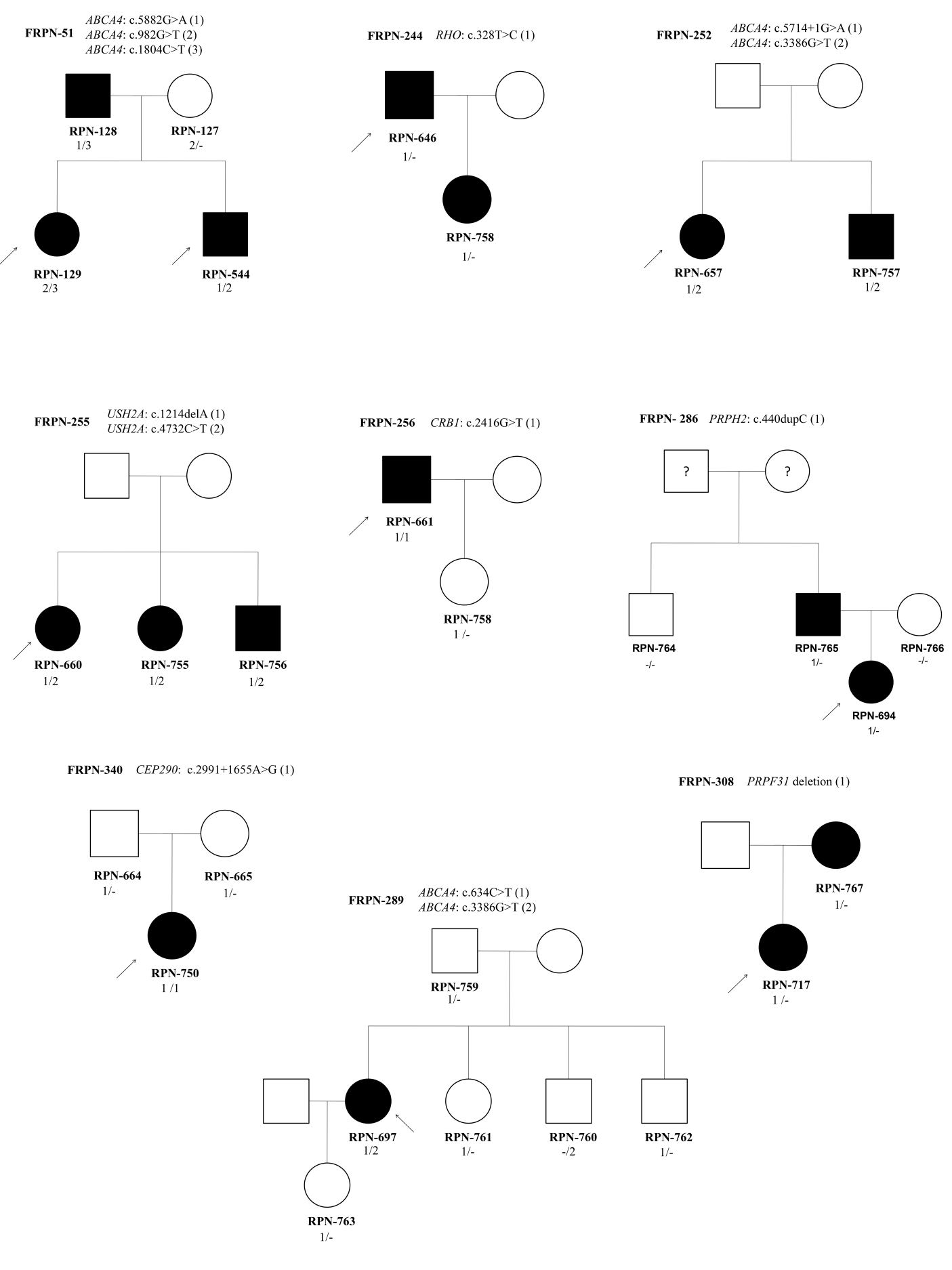
**

**Figure S1. Family pedigrees in which segregation analysis was carried out.** Arrows indicates proband members in each family. FRPN: Family number; RPN: Patient number; ?: Unknown clinic.
